# Fitness Factors and Microbial Behavior in Guarana Beverages

**DOI:** 10.1111/1750-3841.70848

**Published:** 2026-01-14

**Authors:** Dionisio Pedro Amorim‐Neto, Clara Mariana Gonçalves Lima, Jaqueline Sousa Correia, Emilie Lang, Róisín M. Owens, Simone de Nazaré Melo Ramos, Priscilla Efraim, Anderson S. Sant'Ana

**Affiliations:** ^1^ Department of Food Science and Nutrition, Faculty of Food Engineering University of Campinas Campinas SP Brazil; ^2^ Department of Food Engineering and Technology, Faculty of Food Engineering University of Campinas Campinas SP Brazil; ^3^ Department of Chemical Engineering and Biotechnology University of Cambridge Cambridge UK

## Abstract

Although recent studies have reported the presence of a variety of bacteria in guarana products, particularly in beverages from soda fountain machines, investigations into microbial behavior in the guarana production chain remain limited. This study evaluated the fitness and microbial growth of bacterial isolates from guarana‐based products and by‐products. The behavior of seventeen isolates (*Enterococcus hirae* LMQA‐PG1 and LMQA‐PG2, *Klebsiella pneumoniae* LMQA‐PG4, *Klebsiella variicola* LMQA‐PG4.2, *Cronobacter sp* LMQA‐PG3.2, LMQA‐PG4.1, and LMQA‐PG4.2, *Bacillus cereus* LMQA‐PG9.1, LMQA‐PG2.1, and LMQA‐PG3.1, *Enterobacter cloacae* LMQA‐PG3.3, LMQA‐PG3.1, and LMQA‐PG4.1, *Pseudomonas aeruginosa* LMQA‐PG1.2, and LMQA‐PG1.3, *Escherichia hermannii* LMQA‐PG4.3, and *Salmonella sp*. LMQA‐AF3) were assessed under different pH (3 to 7), temperature (2–55°C), and water activity (*a_w_*) (0.500 to 0.919). Most isolates showed high growth performance when cultivated at 25–42°C and pH 6–7, but were inhibited under extreme conditions, including temperatures of 2, 10, and 55°C, acidic pH values of 3‐3.5, and all *a_w_* tested. Several isolates exhibited enzymatic activities with proteolytic and lipolytic spoilage potential, particularly *Bacillus cereus*, and resistance to more than two antimicrobial agents. Based on fitness performance, eight representative isolates (from *Enterococcus*, *Klebsiella*, *Cronobacter*, *Bacillus cereus*, *Enterobacter*, *Pseudomonas*, *Escherichia hermannii*, and *Salmonella*) were then tested in six guarana beverage matrices, three reconstituted guarana‐flavored powdered refreshments, and three ready‐to‐drink guarana refreshments. None of the beverages stored at 30 or 37°C for microbial growth supported bacterial growth, with reductions up to 6 log UFC/mL observed in reconstituted formulations (pH 3.01–3.29).

**Practical Applications:**

This study demonstrated that all brands of reconstituted guarana‐flavored powdered (pH 3.12) and ready‐to‐drink guarana refreshment (pH 3.31) beverages investigated inhibited the microbial growth of bacteria isolated from guarana products and production residues.

## 1 Introduction

The consumption of beverages prepared from fermented and roasted guarana seeds, the fruit of *Paullinia cupana*, dates back to the 18th century among Indigenous communities in the Amazon region of Brazil (Smith and Atroch 2010). This beverage is traditionally made by mixing guarana powder or grated guarana sticks with water to create a drink that embodies Brazilian cultural traditions (Schimpl et al. 2013).

Essentially, the consumption of guarana‐based beverages is associated with their energizing and aphrodisiac properties (Henman 1982). However, the presence of bioactive compounds derived from methylxanthines (especially caffeine) and flavonoids has also been linked to cognitive, anti‐inflammatory, and antioxidant benefits (as reviewed by (Torres et al. 2022)), improving guarana beverages to a level of importance that is not only cultural but also nutritional and functional.

Currently, the destination of guarana powder for the production of beverages can be divided toward the production of carbonated guarana beverages (guarana soda) (Smith and Atroch 2007), as well as two other types of non‐carbonated beverages: reconstituted guarana‐flavored powdered drink and ready‐to‐drink guarana refreshments, both based on guarana extract, also derived from fermented, roasted, and ground seeds. According to Brazilian legislation, refreshments (“refrescos” in Portuguese) are regulated by normative instruction N° 19, dated June 19, 2013. They are defined as “non‐fermented beverages obtained by diluting fruit juice, pulp, or plant extract of their origin in potable water, with or without the addition of sugars” (BRASIL 2019; Rego et al. 2020).

Although recent studies have addressed the culturable microbiological profile of the guarana production chain and its by‐products (Amorim‐Neto et al. 2025; Lima et al. 2025), it is still unclear whether these microorganisms can persist in guarana‐based beverages after processing, a concern underscored by the detection of *Bacillus cereus*, *Pseudomonas aeruginosa*, and *Klebsiella pneumoniae* in guarana powder and sticks, essential ingredients for sodas and other guarana‐derived drinks.

The idea that these beverages may sustain the survival of microorganisms is further reinforced by broader evidence from soft drink dispensing systems, where *E. coli*, *Klebsiella pneumoniae*, and *Staphylococcus epidermidis* have been identified (White et al. 2010). Similarly, Hile et al. (2023) found total coliforms and *E. coli* in 42% and 25% of soda fountain water samples, respectively, highlighting a notable risk. Consistent with these findings, elevated levels of coliforms and heterotrophic bacteria in such systems have also been consistently reported in earlier studies (Chaberny et al. 2006; Godard et al. 2013).

Overall, the evidence raises concerns about post‐process contamination in guarana‐based beverages, while underscoring the lack of studies on microorganisms from guarana's key ingredients and their growth potential in the beverages themselves. For this reason, this study aimed to assess the fitness (its ability to survive under specific environmental conditions, such as substrate availability, physicochemical factors, and the presence of antimicrobials (Jordana‐Lluch et al. 2023)) of bacterial isolates previously obtained from the guarana ingredients production chain across different brands and types of guarana beverage matrices.

## 2 Materials and Methods

### 2.1 Bacterial Isolates Cultivation

Seventeen isolates previously obtained from the guarana production chain, including *Enterococcus hirae* (2, LMQA‐PG1 and LMQA‐PG2), *Klebsiella pneumoniae* (1, LMQA‐PG4), *Klebsiella variicola* (1, LMQA‐PG4.2), *Cronobacter* sp. (3, LMQA‐PG3.2, LMQA‐PG4.1, and LMQA‐PG4.2), *Bacillus cereus* (3, LMQA‐PG9.1, LMQA‐PG2.1, and LMQA‐PG3.1), *Enterobacter cloacae* (3, LMQA‐PG3.3, LMQA‐PG3.1, and LMQA‐PG4.1), *Pseudomonas aeruginosa* (2, LMQA‐PG1.2 and LMQA‐PG1.3), *Escherichia hermannii* (1, LMQA‐PG4.3) (Amorim‐Neto et al. 2025; Lima et al. 2025) and *Salmonella* sp. (1, LMQA‐AF3) were used in these study. For the experiments, bacteria were twice cultivated in tryptic soy broth (TSB) for 24 h and incubated at 37°C, except for *Bacillus cereus*, which was maintained at 30°C. Cultures were then streaked onto tryptic soy agar (TSA) plates and incubated at the respective temperatures. Colonies were subsequently resuspended in 0.85% (w/v) saline solution and adjusted to a 0.5 McFarland standard (ANVISA 2005). This standardized inoculum was used for the subsequent experiments.

### 2.2 Stress Tolerance

For the stress tolerance assays related to the guarana production chain, methodologies adapted from those of Wang et al. (2020), Yamamoto et al. (2021), and Zhang et al. (2022) were employed. In a 96‐well plate, a 1% (v/v) bacterial inoculum was added to 200 µL of TSB and incubated at temperatures ranging from 2 to 55°C to assess growth at various temperatures. For pH tolerance, TSB was adjusted with 1N HCl to pH values of 3, 3.5, 5, 6, 6.5, and 7. To evaluate water activity tolerance, TSB was supplemented with glycerol to achieve water activity levels of 0.919, 0.903, 0.532, and 0.500. Plates were incubated for 24 h for all tests. For pH and water activity tolerance assays, incubation was performed at 30°C for *Bacillus cereus* and 37°C for the other microorganisms. As a control, isolates were inoculated into pure TSB and incubated at their respective optimal temperatures. After incubation, the optical density was measured at a wavelength of 600 nm using a microplate reader. Absorbance data were normalized against the control group and expressed as a percentage of the control group. For each stress condition, isolates were categorized as tolerant (≥ 75%), moderately tolerant (≥ 65% and <75%), low tolerant (≥ 45% and <65%), or non‐tolerant (<45%).

### 2.3 Enzymatic Activity

For the enzymatic activity assays, methodologies adapted from Arslan and Özdemir (2011), Fernández‐Pacheco et al. (2021), and Gou et al. (2021) were used. Briefly, 5 µL of each bacterial inoculum was applied onto TSA plates supplemented with 10% (w/v) skimmed milk powder or 10% (v/v) cashew‐based plant milk to assess protease activity and with 20% (v/v) egg yolk or 1.8% (v/v) soybean lecithin to assess lipase activity. Plates were incubated 24 h at 30°C for *Bacillus cereus* and 37°C for the other microorganisms. After incubation, the diameters of the halos and colonies were measured. Enzymatic activity was determined by calculating the ratio of colony diameter to halo diameter (Pz value). Isolates were categorized as showing negative activity (Pz = 1), low activity (Pz = 0.99–0.70), moderate activity (Pz = 0.69–0.50), or high activity (Pz ≤ 0.50) (Fernández‐Pacheco et al. 2021).

### 2.4 Antimicrobial Disk Diffusion Susceptibility Test

For the disk‐diffusion test, the bacterial inoculum was spread onto Mueller‐Hinton agar plates using a swab, followed by the placement of disks containing ceftazidime (CAZ, 30 µg), tetracycline (TET, 30 µg), oxacillin (OXA, 1 µg), streptomycin (EST, 10 µg), ciprofloxacin (CIP, 5 µg), gentamicin (GEN, 10 µg), erythromycin (ERI, 15 µg), and clindamycin (CLI, 2 µg). The plates were then incubated at 35°C for 24 h. After incubation, the inhibition halos were measured, and isolates were classified as resistant, intermediate, or susceptible to each antimicrobial (CLSI 2016; EUCAST 2025).

### 2.5 Microbial Growth (δ) of Bacterial Isolates From the guarana Production Chain in Different Beverage Matrices

Eight isolates, each representing a different genus, were selected for the microbial growth assay in beverage matrices. Three leading brands of commercial guarana‐flavored powdered drinks (prepared according to the manufacturer's instructions) and three ready‐to‐drink guarana refreshments were used. Before the tests, all beverages were inoculated onto selective media to confirm the absence of the target microorganisms. In sterile 15 mL Falcon tubes, 10 mL of each beverage was separately dispensed and inoculated to achieve a final bacterial concentration of approximately 6 log_10_ CFU/mL. Tubes were incubated for 6 h at 30°C for *Bacillus cereus* and at 37°C for the other bacteria, which correspond to their respective optimal growth temperatures ranges (Stott et al. 2015). Bacterial counts were performed using the spread plate method at 0, 3, and 6 h of incubation. Selective media were used for microbial enumeration: Harlequin *Cronobacter* Isolation Agar for *Cronobacter* sp. (Neogen, United Kingdom), Mannitol Egg Yolk Polymyxin (MYP) Agar for *B. cereus* (Betec‐Sigma‐Aldrich Brazil Ltda., Duque de Caxias, Brazil), KF *Streptococcus* Agar Medium (Neogen, United Kingdom) for *Enterococcus*, xylose lysine deoxycholate (XLD) Agar for *Salmonella* (Kasvi, Shanghai, China), MacConkey Agar for *Escherichia* and *Klebsiella* (Merck, Germany), *Pseudomonas* Cetrimide Agar glycerol pseudomonas agar (GSP) for *Pseudomonas* (Merck, Germany). Plates were incubated for 24 h at 30°C for *Bacillus cereus* and 37°C for the other bacteria. Data were expressed as log_10_ CFU/mL, with a detection limit (DL)of 2 log_10_ CFU/mL.

The microbial growth (δ, log_10_) of the selected bacterial isolates in each guarana beverage matrix was determined by Equation 1, calculated as the difference between microbial counts (log_10_ CFU/mL) at the final time point (3 or 6 h) and the initial time point (time zero). Beverages were considered supportive of bacterial growth when positive δ values were observed, indicating an increase in bacterial population in the reconstituted guarana beverage matrix, while negative δ values indicate a population decrease (EURLLM 2023; AFSSA 2003). The DL threshold of the method corresponds to the maximum loss of detectable viable cells, being approximately –6 for *Enterococcus hirae*, *Klebsiella pneumoniae*, *Cronobacter sp*., *Enterobacter cloacae*, for *Pseudomonas aeruginos*a, *Escherichia hermannii*, and *Salmonella sp., and* –5 for *Bacillus cereus*.

(1) $$\delta =logN_{f}-logN_{0}$$

where *N_f_* and *N_0_* indicate the final and initial counts (log_10_ CFU/mL) over the in‐beverage microbial growth assays.

### 2.6 Physico‐Chemical Analysis

The pH was measured with a calibrated portable digital meter (AKSO, model AK 103), and Brix values were obtained using a handheld refractometer (RZ, model RZ120).

### 2.7 Statistics

All tests were performed in triplicate except for the in‐beverage microbial growth assay, which was performed in duplicate. Data were subjected to analysis of variance (ANOVA), followed by honestly significant difference (HSD) tests, principal component analysis (PCA), Student's t‐test for comparisons between two groups, and Tukey's test for comparisons among three or more groups. All statistical analyses were performed using RStudio software v.4.1.2 (R core team 2021).

## 3 Results and Discussion

**FIGURE 1:**
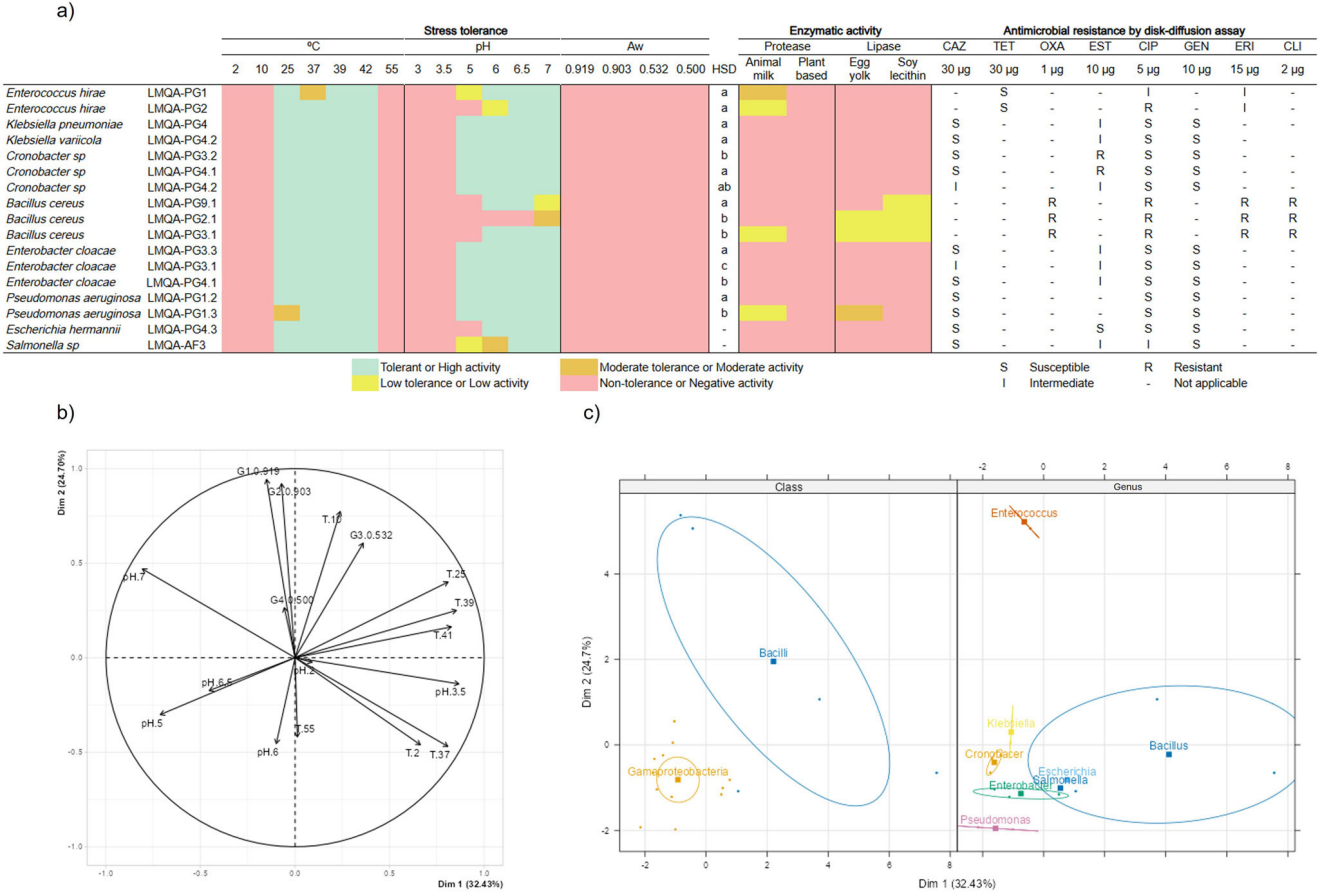
Summary of fitness profiles based on (a) stress tolerance and enzymatic activity in guarana‐associated strains. Principal Component Analysis of Stress Tolerance in Bacterial isolates from the Guarana Production Chain. (b) Correlation circle of the PCA. (c) Correlation taxonomic stratification.

Initially, the fitness of seventeen bacterial isolates from the guarana production chain was assessed through in vitro experiments (Figure 1a). Most isolates tolerated temperatures between 25–42°C, except for *E. hirae* (LMQA‐PG1) and *P. aeruginosa* (LMQA‐PG1.3), which grew under neutral to mildly acidic pH conditions (pH 6–7). At pH 5, on the other hand, isolates belonging to the genera *Klebsiella*, *Cronobacter*, *Enterobacter*, and *Pseudomonas* were found to be tolerant. None of the isolates resisted extreme temperatures (2, 10, or 55°C), highly acidic pH (3–3.5), or low water activity (*a_w_* 0.500–0.919). The choice of variables reflects the characteristics of the climate where guarana is cultivated, the extract production process, the guarana wastes such as “casquilho” and bagasse, and various guarana‐based products and residues, including guarana powder, guarana stick, guarana syrup (Amorim‐Neto et al. 2025; Nazeré 1998; Tavares et al. 2005), guarana soda (Barac et al. 2015; Helena et al. 2007), reconstituted guarana‐flavored powdered drink and ready‐to‐drink guarana refreshment (Table 1).

**TABLE 1:** Physicochemical properties^*^ of reconstituted guarana powder and ready‐to‐drink guarana refreshments beverages.

—	—	pH	Water activity	Brix
Reconstituted guarana‐flavored powdered refreshments	I	3.08 ± 0.01^b^	0.998 ± 0.001^ab^	1.8^b^
	II	3.29 ± 0.01^a^	0.997 ± 0.001^b^	1.8^a^
	III	3.01 ± 0.01^c^	0.999 ± 0.000^a^	1.6^c^
Ready‐to‐drink guarana refreshments	IV	3.29 ± 0.01^b^	0.999 ± 0.001^a^	7.4^b^
	V	3.31 ± 0.02^ab^	0.997 ± 0.001^ab^	7.6^c^
	VI	3.34 ± 0.02^a^	0.996 ± 0.001^b^	9.6^a^

*Notes*: ^*^Results are expressed as mean ± standard deviation.

^a–c^Different lowercase letters in the same column indicate statistically significant differences between refreshments brands, according to Tukey's test (*p* < 0.05), for the same beverage category.

For enzymatic assays, *E. hirae* (LMQA‐PG1, LMQA‐PG2), *B. cereus* (LMQA‐PG3.1), and *P. aeruginosa* (LMQA‐PG1.3) exhibited low to moderate proteolytic activity against animal‐based proteins only. No activity was observed against the plant‐based milk. Most *B. cereus* isolates showed lipolytic activity on both lipid substrates, while *P. aeruginosa* (LMQA‐PG1.3) showed activity only on egg yolk. Protease and lipase activities from foodborne bacteria are well‐established as primary drivers of spoilage in foods. Their heat stability and broad activity range, especially in *B. cereus* and *Pseudomonas aeruginosa* species, make them particularly problematic for food safety and shelf‐life (Arslan et al. 2011; Sun et al. 2025).

Thereafter, a Principal Component Analysis was performed (Figures 1b and 1c). The first two principal components, Dim 1 and Dim 2, explained 57.13% of the total variability, accounting for 24.7% and 32.43%, respectively. Some correlation patterns were evident: slightly acidic pH values were negatively associated with both components, while osmotic stress and intermediate temperatures showed positive correlations (Figure 1b). Additionally, Gram‐negative isolates were negatively correlated with both Dim 1 and Dim 2 (Figure 1c). In summary, the Gram‐negative isolates exhibit lower tolerance to temperature and osmotic stress, but they perform better at neutral or slightly acidic pH levels. In this context, previous studies have reported high contamination rates of carbonated soft drinks with several Gram‐negative bacteria, like *Pseudomonas*, *Salmonella*, and coliforms (Akond et al. 2009).

The antimicrobial resistance profile (Figure 1a) was evaluated using the disk‐diffusion method. One *Enterococcus* isolate was resistant to ciprofloxacin, and both were intermediate to erythromycin. *Klebsiella* showed intermediate resistance to streptomycin. Two out of three *Cronobacter* isolates were resistant to streptomycin. *Bacillus cereus* was resistant to all tested antimicrobials. All *Enterobacter* isolates showed intermediate resistance to streptomycin. *Pseudomonas aeruginosa* and *E. hermannii* were susceptible to all antimicrobials tested, while *Salmonella* exhibited intermediate resistance to both streptomycin and ciprofloxacin. Other studies involving low‐water activity foods have shown similar resistance profiles in *Cronobacter* isolated from powdered milk (Fei et al. 2022), *Salmonella* from chocolate granules (Aguilar‐Vázquez et al. 2022), and *Bacillus cereus* from infant formula powder (Fei et al. 2021). Taken together, these findings are concerning, as the foods from which the bacteria were isolated, such as guarana powder, for example, can be consumed raw or used in the preparation of juices, refreshments, and other products, potentially carrying resistant or intermediately resistant microorganisms.

Finally, based on the tests previously described, eight isolates, one from each genus, were selected for microbial growth assays to assess whether guarana beverage matrices from six different brands supported microbial growth at their optimal incubation temperatures ranges (Stott et al. 2015). For this, the bacterial inoculum was added to reconstituted guarana‐flavored powdered drinks (brands I, II, and III) and ready‐to‐drink guarana refreshments (brands IV, V, and VI). None of the brands in both beverage categories were able to support microbial growth (negative δ) at either 3 or 6 h in the eight isolates challenged.

Reconstituted guarana‐flavored powdered drink (highlighting Brand I, pH 3.08 and 1.8 Brix, *a_w_* 0.998, Table 1 and Table 2) showed stronger and earlier inhibition, with many isolates below the DL even at 3 h (*K. pneumoniae* LMQA‐PG4 and *P. aeruginosa* LMQA‐PG1.3). Regarding the ready‐to‐drink guarana refreshments, growth inhibition increased over time, with results below the DL more frequently observed after 6 h, especially in sample V (pH 3.31, 7.6 Brix, and *a_w_* 0.997, Table 1 and Table 2), except for the isolates *E. hirae* LMQA‐PG1, *K. pneumoniae* LMQA‐PG4, and *B. cereus* LMQA‐PG3.1. On the other hand, *P. aeruginosa*, *E. hermannii*, and *Salmonella sp*. were among the most sensitive isolates, often undetectable in multiple beverage matrices.

**TABLE 2:** Microbial growth^+^ (δ) of bacterial isolates in the reconstituted guarana‐flavored powdered refreshments or ready‐to‐drink guarana refreshments stored at the optimal growth temperature^&^ for each microorganism.

Reconstituted guarana‐flavored powdered refreshments																								
Exposure time	3 h												6 h											
Commercial brands	I				II				III				I				II				III			
*Enterococcus hirae* LMQA‐PG1	−5.7	±	0.1	*c*	−2.4	±	0.1	*a*	−4.1	±	0.1	*b*	<DL	±	NA	NA	−4.4	±	0.1	^*^	−5.4	±	0.1	^NA^
*Klebsiella pneumoniae* LMQA‐PG4	<DL	±	NA	NA	−2.8	±	0.1	ns	−2.9	±	0.1	NA	<DL	±	NA	NA	−3.5	±	0.1	^ns^	−4.0	±	0.4	^NA^
*Cronobacter sp* LMQA‐PG4.1	−4.7	±	0.1	*b*	−1.6	±	0.0	*a*	−5.2	±	0.3	*b*	<DL	±	NA	NA	−2.7	±	0.2	^NA^	<DL	±	NA	NA
*Bacillus cereus* LMQA‐PG3.1	−2.1	±	0.1	*b*	−1.0	±	0.1	*a*	−1.0	±	0.0	*a*	−1.9	±	0.1	b	−1.0	±	0.0	^a^	−1.0	±	0.0	a
*Enterobacter cloacae* LMQA‐PG3.1	−3.0	±	0.2	*a*	−4.4	±	0.1	*b*	−4.7	±	0.1	*b*	−4.2	±	0.1	NA	<DL	±	NA	^NA^	<DL	±	NA	NA
*Pseudomonas aeruginosa* LMQA‐PG1.3	<DL	±	NA	NA	<DL	±	NA	NA	<DL	±	NA	NA	<DL	±	NA	NA	<DL	±	NA	^NA^	<DL	±	NA	NA
*Escherichia hermannii* LMQA‐PG4.3	−5.2	±	0.5	*a*	−4.4	±	0.4	*a*	−3.7	±	0.1	*a*	<DL	±	NA	NA	−5.0	±	0.1	^NA^	<DL	±	NA	NA
*Salmonella sp* LMQA‐AF3	−5.7	±	0.1	*a*	−4.1	±	0.1	*a*	−4.1	±	0.2	*a*	<DL	±	NA	NA	<DL	±	NA	^NA^	<DL	±	NA	NA
**Ready‐to‐drink guarana refreshments**																								
**Exposure time**	**3 h**												**6 h**											
**Commercial brands**	**IV**				**V**				**VI**				**IV**				**V**				**VI**			
*Enterococcus hirae* LMQA‐PG1	−4.3	±	0.1	*b*	−3.1	±	0.1	*a*	−4.3	±	0.1	*b*	−5.6	±	0.2	a	−4.8	±	0.8	^a^	−4.8	±	0.1	^a^
*Klebsiella pneumoniae* LMQA‐PG4	−1.6	±	0.2	*a*	−3.8	±	0.1	*b*	−3.1	±	0.1	*b*	−3.1	±	0.1	NA	NA	±	NA	^NA^	<DL	±	NA	^NA^
*Cronobacter sp* LMQA‐PG4.1	−4.5	±	0.1	*a*	−5.0	±	0.1	*ab*	−5.4	±	0.1	*b*	<DL	±	NA	NA	<DL	±	NA	^NA^	<DL	±	NA	^NA^
*Bacillus cereus* LMQA‐PG3.1	−1.3	±	>0.0	*a*	−1.4	±	0.1	*a*	−1.2	±	0.1	*a*	−2.5	±	0.1	b	−1.3	±	0.3	^a^	−1.3	±	0.2	^a^
*Enterobacter cloacae* LMQA‐PG3.1	−3.9	±	0.0	*a*	−4.6	±	0.1	*b*	−3.6	±	0.0	*a*	<DL	±	NA	NA	<DL	±	NA	^NA^	−4.4	±	0.4	^NA^
*Pseudomonas aeruginosa* LMQA‐PG1.3	<DL	±	NA	*NA*	<DL	±	NA	NA	<DL	±	NA	NA	<DL	±	NA	NA	<DL	±	NA	^NA^	<DL	±	NA	^NA^
*Escherichia hermannii* LMQA‐PG4.3	−4.4	±	0.1	*a*	−4.2	±	0.2	*a*	−5.1	±	0.0	*b*	<DL	±	NA	NA	<DL	±	NA	^NA^	<DL	±	NA	^NA^
*Salmonella sp* LMQA‐AF3	−3.9	±	0.9	*a*	−4.7	±	0.1	*a*	−3.7	±	0.1	*a*	−5.0	±	0.1	ns	<DL	±	NA	^NA^	−4.9	±	0.1	^NA^

*Notes*: ^+^Microbial growth (**δ**, log_10_) was calculated as the difference between final (3 or 6 h) and initial (0 h) microbial counts. Experiments were performed in duplicate, and results are expressed as mean ± standard deviation.

^&^All microorganisms were incubated at 37°C, except *B. cereus*, which was incubated at 30°C.

^a‐c^ Different lowercase letters in the same line indicate statistically significant differences between refreshments brands, according to Tukey's test (*p* < 0.05), for the same time point and bacterial species.

For groups where one sample was below the detection limit (DL), the t‐test was applied to the two remaining samples. In this case, the symbol * in a line indicates statistically significant differences (*p* < 0.05) between refreshments brands or ns (*p* > 0.05) for the same time point and bacterial species.

A positive δ value signifies an increase in the bacterial population within the reconstituted guarana‐flavored powdered refreshments or ready‐to‐drink guarana refreshments. Conversely, a negative δ value indicates a decrease in the bacterial population.

NA not applicable.

To the best of the author's knowledge, no studies have assessed the microbial growth of microorganisms isolated from guarana products and by‐products in refreshments, making comparisons with literature difficult; therefore, a broader perspective was adopted, including studies on cola and other fruit matrices, although such reports remain scarce. In this context, a similar phenomenon was observed in diet cola (pH 2.44), where *B. pseudomallei* (a Gram‐negative bacterium) showed no growth within 2 hours, and *Salmonella* and *E. coli* were inhibited within 48 hours (Sheth et al. 1988; Wuthiekanun et al. 2020). The antimicrobial effect of acidification has also been reported in açaí juice, where a low pH (∼4.3) enhanced the inactivation of *E. coli* O157:H7 and *Salmonella spp*. under high‐pressure processing, while raising the pH to 5.5 markedly reduced treatment efficacy (Gouvea et al. 2020). Conversely, *B. cereus* (LMQA‐PG3.1), a spore‐forming, toxin‐producing bacterium (Logan and Vos 2015), remained detectable in all beverage brands; however, the mechanisms of tolerance in both vegetative cells and spores require further investigation.

## 4 Conclusion

In summary, the bacterial isolates from the guarana production chain characterized in this study tolerated only moderate temperatures (25–42°C) and mildly acidic to neutral pH levels (5–7). Regarding enzymatic activity, *Bacillus cereus* showed the highest lipolytic and proteolytic performance among the tested isolates. In addition, several isolates exhibited resistance or intermediate resistance to more than two antibiotics, indicating potential antimicrobial tolerance. Finally, even when using the isolates that performed best in the preliminary screenings, none of the isolates from the eight representative genera were able to grow in guarana‐based beverages, either reconstituted powdered drinks or ready‐to‐drink formulations, when stored at the typical temperatures used for isolating these microorganisms.

## Author Contributions

**Dionisio Pedro Amorim‐neto**: conceptualization, investigation, writing – original draft, writing – review and editing, visualization, validation, methodology, formal analysis, data curation. **Clara Mariana Gonçalves Lima**: conceptualization, investigation, writing – original draft, writing – review and editing, visualization, validation, methodology, formal analysis, data curation. **Jaqueline Sousa Correia**: investigation, writing – original draft, validation, methodology, visualization, formal analysis. **Emilie Lang**: investigation, writing – original draft, writing – review and editing, visualization, validation, methodology, formal analysis. **Róisín M. Owens**: writing – original draft, writing – review and editing, visualization, validation, formal analysis. **Simone de Nazaré Melo Ramos**: conceptualization, investigation, writing – original draft, writing – review and editing, visualization, validation, methodology, formal analysis, supervision. **Priscilla Efraim**: investigation, funding acquisition, writing – original draft, writing – review and editing, visualization, validation, methodology, formal analysis, data curation, supervision, project administration, resources. **Anderson S. Sant'ana**: conceptualization, investigation, writing – original draft, writing – review and editing, visualization, methodology, validation, formal analysis, supervision, resources, project administration, funding acquisition.

## Conflicts of Interest

The authors declare no conflicts of interest.
